# Supplementary material for: Dynamic transcriptomic profiles of zebrafish gills in response to zinc supplementation
Source: BMC Genomics. 2010 Oct 11;11:553. doi: 10.1186/1471-2164-11-553 (PMC3091702; doi:10.1186/1471-2164-11-553)
Supplement: Additional file 2 — Interactive Direct Interaction Network representing the molecular interactions between zinc, copper, iron, calcium and proteins encoded by transcripts changed by zinc supplementation. Mini web-site containing index.html and hyperlinked pages in subdirectory describing a Direct Interaction Network automatically generated based on curated interactions contained within the proprietary PathwayArchitect database. Ovals represent proteins and the circles symbolize metal ions. Objects are coloured by their abundance in zebrafish at the time-point they were significantly different from the control is a scale from -4 fold (dark green) to +4 fold (dark red). Where significant differences were found at more than one time-point, the colour overlay shows expression at the first instance. Dark blue squares denote 'binding', and light blue squares 'expression'; green squares stand for 'regulation', green diamonds for 'metabolism', and green circles for 'promoter binding'. Arrow heads indicate directionality of the interaction where annotated. All nodes and edges can be further interrogated by selecting the relative area of the image. [file 1471-2164-11-553-S2.zip › PathwayArchitect Zn xs DIN/460460.html]

# REGULATION:

|  |  |
| --- | --- |
| Type | REGULATION |
| Effect | Positive |


---

|  |  |
| --- | --- |
| Score | 0 |


---

|  |  |
| --- | --- |
| Reference Count | 10 |


---

|  |  |
| --- | --- |
| Mechanism | Unknown |


---

|  |  |
| --- | --- |
| Reference:0 || Sentence | "Ethanol administration counteracts the protective effect of zinc on iron concentration and total iron binding capacity, while the favorable action of zinc is maintained in respect of hematocrit, hemoglobin concentration and transferrin saturation index." |
| PMID | 2616387 |
| Year | 1989 |
| Species | Rat |
| Journal | Pol J Pharmacol Pharm |
| RefScore | 1 |
| Source | PArchNLP |
  |
|  |  |


---

|  |  |
| --- | --- |
 Reference:1 || Sentence | "Interestingly, our current data indicate that zinc upregulates DMT1 protein and mRNA expression and also pH-dependent iron uptake." |
| PMID | 11684086 |
| Year | 2001 |
| Species | Human |
| Journal | FEBS Lett |
| RefScore | 0 |
| Source | PArchNLP |
  ||  |  |


---

|  |  |
| --- | --- |
 Reference:2 || Sentence | "The protective effects of ZnCl2 may be due to lower iron uptake into hepatocytes and by the induction of zinc metallothionein, which can serve as a scavenger for oxygen radicals." |
| PMID | 1917562 |
| Year | 1991 |
| Species | Rat |
| Journal | Histochem J |
| RefScore | 0 |
| Source | PArchNLP |
  ||  |  |


---

|  |  |
| --- | --- |
 Reference:3 || Sentence | "When the exposures were analyzed as continuous variables, the adjusted RRs (CI) were 1.66 (1.03-2.68) for 100 microg/dl iron increase, 1.17 (1.01-1.36) for 10% TS increase, 1.98 (1.12-3.50) for 100 microg/dl copper increase, and 0.57 (0.16-1.96) for 100 microg/dl zinc increase." |
| PMID | 15036223 |
| Year | 2004 |
| Species | Human |
| Journal | Ann Epidemiol |
| RefScore | 1 |
| Source | PArchNLP |
  ||  |  |


---

|  |  |
| --- | --- |
 Reference:4 || Sentence | "Overexpression of Cu/Zn-SOD and Mn-SOD also partially protected E47 cells from the increase in reactive oxygen production and lipid peroxidation and the loss of mitochondrial membrane potential induced by AA and iron." |
| PMID | 14578853 |
| Year | 2003 |
| Species | Human |
| Journal | Hepatology |
| RefScore | 1 |
| Source | PArchNLP |
  ||  |  |


---

|  |  |
| --- | --- |
 Reference:5 || Sentence | "This study reports the effect of paraquat (PQ) on concentrations of four elements (Cu, Fe, Mg, Zn) in lung, kidney, spleen, liver, and heart of male osteogenic disorder Shionogi (ODS) rats, a strain not able to synthesize vitamin C." |
| PMID | 11820502 |
| Year | 2002 |
| Species | Rat |
| Journal | J Toxicol Environ Health A |
| RefScore | 1 |
| Source | PArchNLP |
  ||  |  |


---

|  |  |
| --- | --- |
 Reference:6 || Sentence | "Serum Zn varied with dietary Zn with a small negative influence from the interaction between Cu and Fe." |
| PMID | 10395600 |
| Year | 1999 |
| Species | Rat |
| Journal | J Nutr |
| RefScore | 1 |
| Source | PArchNLP |
  ||  |  |


---

|  |  |
| --- | --- |
 Reference:7 || Sentence | "In summary, Fe status was minimally influenced by dietary Zn or Cu, and Fe intakes 10-fold greater than required did not induce overt oxidative stress in female rats." |
| PMID | 10395600 |
| Year | 1999 |
| Species | Rat |
| Journal | J Nutr |
| RefScore | 2 |
| Source | PArchNLP |
  ||  |  |


---

|  |  |
| --- | --- |
 Reference:8 || Sentence | "In addition, Zn deficiency caused changes in the levels of Zn and other trace elements such as Ca, Al, S, Fe, and Cl and vitamin C." |
| PMID | 15103203 |
| Year | 2004 |
| Species | Rat |
| Journal | Ophthalmic Res |
| RefScore | 1 |
| Source | PArchNLP |
  ||  |  |


---

|  |  |
| --- | --- |
 Reference:9 || Sentence | Zn increased on d 1 and 2, and Fe increased on d 2. |
| Year | 2006 |
| PMID | 16388104 |
| Species | Mouse |
| Journal | Biol Trace Elem Res |
| RefScore | 2 |
| Source | PArchNLP |
  |


---

|  |  |
| --- | --- |
